# Intergenerational Chain of Violence, Adverse Childhood Experiences, and Elder Abuse Perpetration

**DOI:** 10.1001/jamanetworkopen.2024.36150

**Published:** 2024-09-27

**Authors:** Chie Koga, Taishi Tsuji, Masamichi Hanazato, Atsushi Nakagomi, Takahiro Tabuchi

**Affiliations:** 1 Research Center for Advanced Science and Technology, University of Tokyo, Tokyo, Japan; 2 Institute of Health and Sport Sciences, University of Tsukuba, Tokyo, Japan; 3 Center for Preventive Medical Sciences, Chiba University, Chiba, Japan; 4 Department of Cancer Epidemiology, Cancer Control Center, Osaka International Cancer Institute, Osaka, Japan; 5 Division of Epidemiology, School of Public Health, Tohoku University, Graduate School of Medicine, Sendai, Japan

## Abstract

**Importance:**

It is widely known that individuals with adverse childhood experiences (ACEs) have an increased risk of abusing their own children, thereby perpetuating the cycle of violence. However, the association between ACEs and elder abuse perpetration has not been fully examined.

**Objective:**

To examine the association between ACEs and elder abuse and the mediating factors.

**Design, Setting, and Participants:**

This cross-sectional study used data collected via the self-administered Japan COVID-19 and Society Internet Survey from September 12 to October 19, 2022. Men and women aged 20 to 64 years who responded to related questions were included. Data were analyzed from July 2023 to April 2024.

**Exposures:**

ACEs, defined as the experience of any of 7 items—interpersonal loss (parental loss and parental divorce), family psychopathology (parental mental disease and violence in family), abuse (physical and psychological abuse), and neglect—before the age of 18 years.

**Main Outcomes and Measures:**

The primary outcome was the perpetration of physical and/or psychological abuse against an older person (aged ≥65 years) self-reported via questionnaire. The direct and indirect effect estimates were determined using logistic regression analyses.

**Results:**

Of a total of 13 318 participants (mean [SD] age, 41.1 [12.1] years; 6634 female [49.8%]), 1133 (8.5%) reported perpetrating violence against older adults. Compared with individuals without ACEs, the odds ratios (ORs) for perpetrating violence were 3.22 (95% CI, 2.74-3.79) for those with 1 ACE and 7.65 (95% CI, 6.41-9.13) for those with 2 or more ACEs. In the mediation analysis, factors with large indirect effect estimates included depression (OR, 1.13; 95% CI, 1.11-1.14; proportion mediated [PM], 18.6%), mental illness other than depression (OR, 1.12; 95% CI, 1.10-1.14; PM, 17.3%), and self-rated health (OR, 1.04; 95% CI, 1.03-1.05; PM, 6.0%).

**Conclusions and Relevance:**

These findings suggest that intergenerational cycles of violence may extend to any vulnerable group, not only children but also older adults. Further research into the prevention of ACEs and breaking these cycles of violence is warranted.

## Key Points

**Question:**

Do individuals with adverse childhood experiences (ACEs) have an increased risk of perpetrating violence against older adults (ie, those aged ≥65 years)?

**Findings:**

In this cross-sectional study of 13 318 adults aged 20 to 64 years, a higher number of ACEs was associated with the perpetration of violence against older adults. Compared with adults with no ACEs, those with 1 ACE had more than 3 times the risk of violence, and those with 2 or more ACEs had nearly 8 times the risk of violence.

**Meaning:**

These findings suggest that the intergenerational cycle of violence may extend to elder abuse, in addition to child abuse, and additional research regarding the prevention of ACEs is warranted.

## Teaser

This cross-sectional study examines the association between adverse childhood experiences and elder abuse and factors that may mediate this association.

## Introduction

It has been reported that abuse is intergenerational.^1^ Therefore, individuals who have experienced adverse childhood experiences (ACEs) are at an increased risk of abusing their children—that is, continuing the cycle of violence. The concept of ACEs, which includes loss of interpersonal relationships, family psychopathology, abuse, and neglect, is more likely to be found among children living in poverty.^2^ It is well-established that ACEs are associated with a variety of adverse health outcomes.^3^ Parents with ACEs reported a higher likelihood of physically aggressive behavior toward their children than those without ACEs. For example, a cross-sectional study by Cuartas et al^4^ involving 11 759 Colombian mothers showed a higher propensity for physical punishment (eg, hitting with objects and spanking) among mothers who had ACEs. In a cross-sectional study involving 679 fathers from Sweden and Finland, Ellonen et al^5^ showed that having ACEs was associated with using punishment in parenting. In Japan, an intergenerational chain of abuse has also been reported in several fields, such as psychology and sociology.^6,7^ Preventing these corporal punishments from occurring is important because they have long-term negative outcomes and undermine the normal growth and well-being of children.^8,9^ A meta-analysis^3^ reported that the outcomes of ACEs were wide-ranging and that increased numbers of ACEs were associated with higher odds ratios (ORs) for adverse psychological or behavioral outcomes than for medical outcomes. Furthermore, there are many studies^10,11,12^ showing that corporal punishment and verbal abuse are associated with delinquency, aggression, poor mental health, and other negative outcomes in adolescents.

The intergenerational cycle of violence has several causes; however, corporal punishment is the most frequently studied. For example, Lansford et al^13^ examined 336 mother-child dyads and reported that corporal punishment is culturally acceptable as a parenting strategy. However, other studies^8^ have shown that corporal punishment, even when culturally acceptable, is associated with adverse outcomes in children. Moreover, this linkage might be explained by the social learning theory, which states that observation and modeling play a major role in how and why individuals learn.^14^ Muller et al^15^ revealed that the intergenerational transmission of corporal punishment fits the social learning model better than temperament. Another study by Taylor et al^16^ found that mothers’ endorsement of domestic violence increases the frequency of hitting their children. In addition to individual factors, community factors may also be associated with this consequence. Cuartas et al^17^ conducted a cross-sectional study involving 1209 children and their mothers in Colombia and found that neighborhood violent crimes, such as homicides and personal injuries, were associated with aggressive discipline. That article discussed this factor as a consideration of the possibility that external threats may inhibit family well-being.

Furthermore, some studies have examined the association between ACEs and being the recipient of elder abuse. Asyraf et al^18^ studied cross-sectional data of 1984 participants aged 60 to 93 years in Kuala Lumpur, Malaysia, and reported that ACEs were significantly associated with experiencing abuse in later life. Moreover, Zhang^19^ studied the cross-sectional data of 8436 older Chinese adults and revealed that ACEs acted as a moderating factor in the association between elder abuse and depressive symptoms. Therefore, those who experienced moderate ACEs were more likely to cope better with elder abuse and were at a lower risk of depression. In contrast, another previous study by Fu et al^20^ using cross-sectional data of 1002 Chinese older adults showed that the association of a higher risk of depression among those with a higher number of ACEs was partially mediated by elder abuse. These results must be validated using data from larger sample sizes and different populations, even though the mechanisms are not yet clear.

However, the association between ACEs and elder abuse perpetration, which is a longer intergenerational link, has not been fully examined, and its mechanism has not been clarified. A previous study^11^ reported that if corporal punishment is associated with general aggressive tendencies in adulthood, this aggression may also appear in relationships with family members, especially with children and spouses. Other studies^21,22,23,24^ found that experiences of corporal punishment were associated with increased likelihood of violence against a partner or spouse. If the intergenerational linkage of abuse is a sociocultural and even community factor, there may be an association between experiencing ACEs and perpetrating elder abuse. Examining these associations will not only clarify the negative outcomes of ACEs on later life but also contribute to the prevention of elder abuse. This study aimed to examine the association between ACEs and elder abuse perpetration and its mediating factors.

## Methods

### Study Setting and Participants

This cross-sectional study used data from a self-administered online survey conducted by the Japan COVID-19 and Society Internet Survey (JACSIS) from September 12 to October 19, 2022. We have included responses to the questions on elder abuse and ACEs. The JACSIS is a project that conducts a survey on lifestyle, health, and social and economic activities, including COVID-19, to provide evidence to protect the health and social activities of the Japanese population. The JACSIS 2021 survey was approved by the Ethics Committee of the Osaka International Cancer Institute (approved on June 19, 2020), and this study was conducted according to the guidelines of the Declaration of Helsinki.^25^ Informed consent was obtained from the participants by the internet survey company. Consent was obtained by filling out a checkbox, clearly indicating the outline of the study, responsible party, contact person, handling of personal information, correspondence, and how to refuse participation.

**Figure 1.**
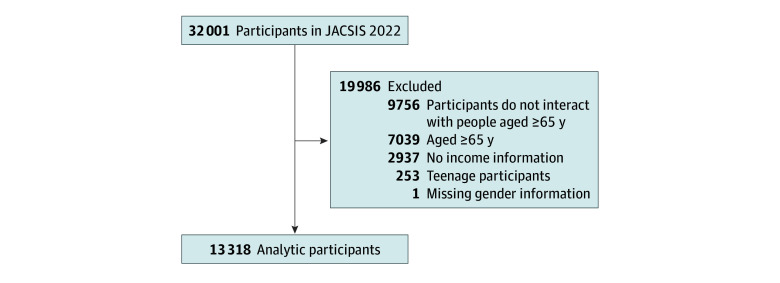
Flow Chart of Study Participants JACSIS indicates Japan COVID-19 and Society Internet Survey.

Although race cannot be specified in this study, the total number of foreign nationals living in Japan is 2.75% of the total population, and most of the participants in this study are considered to be Japanese nationals.^26^ We reserved responses from 32 000 participants (response rate, 65.7%). We excluded participants whose sex, age, and income could not be confirmed; those who reported an error; and those who did not interact with adults aged 65 years or older in general. Furthermore, those who were older than 65 years and those whose answers to questions about abuse and ACEs were missing were excluded (Figure 1).

### Dependent Variable: Perpetration Against Older Adults (Elder Abuse)

The outcome variable was the perpetration of abuse against older adults (aged ≥65 years). Elder abuse was measured in 2 dimensions (physical and psychological) using a self-reported questionnaire developed in collaboration with individuals from multiple fields.^27,28,29,30,31,32^ Because no definition to measure perpetration of elder abuse currently exists, we asked the respondents to respond to a questionnaire with specific behaviors and feelings of abuse. For physical abuse, the participants were asked whether they had engaged in “violence such as punching, kicking, throwing things, or cooping up oneself against older adult (including family members) over 65 years of age.” For psychological abuse, the participants were asked whether “self-esteem damaging actions such as verbal abuse, sarcasm, or ignoring older adult (including family members) over 65 years of age were committed by themselves.” The answers were as follows: (1) first time in the last 2 months, (2) first time in the last 1 year, (3) not first time but first in the last 1 year, (4) not in the last 1 year but before 1 year, and (5) never. Those who checked 5 were considered to have no perpetration against older adults; other participants were deemed to have a perpetration against older adults.

### Independent Variable: ACEs

ACEs were independent variables. The participants were assessed using a self-report questionnaire. This questionnaire comprises 7 questions asking whether the participants had experienced the following events before the age of 18 years: interpersonal loss (parental loss and parental divorce), family psychopathology (parental mental illness and violence in family), abuse (physical and psychological abuse), and neglect.^2^ These experiences were examined for predictive validity according to previous studies that found an association with health issues.^33,34^ The total number of ACEs was then calculated. The participants were grouped into the following categories: 0, 1, or 2 or more ACEs. In the mediation analysis, ACE is calculated using continuous values; in all other analyses, ACE is considered as categorical variables.

### Mediators

**Figure 2.**
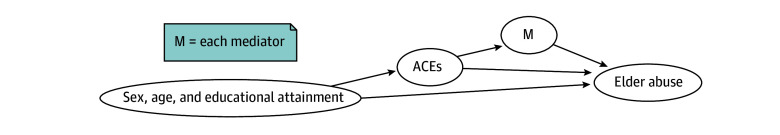
Directed Acyclic Graph ACEs indicates adverse childhood experiences.

Mediators that were hypothesized to occur after exposure to ACEs were considered from previous research.^1,27^ To consider the factors for various areas, 9 basic sociodemographic and health-related factors were analyzed. Sociodemographic factors included living arrangement (living with someone or living alone), employment status (working or unemployed), marital status (unmarried or married), and equivalent income (middle or high income or low income). For health-related factors, we considered self-rated health (not good or good), depression (never or having depression in the past or present), psychological disorders other than depression (never or having these disorders in the past or present), alcohol consumption (currently not drinking or currently drinking), and frequency of going out (which means not homebound) at least once a month (no or yes). We used dichotomized variables based on previous studies.^35^ Directed acyclic graphs illustrating the scenarios are shown in Figure 2. In this study, the temporal mechanisms between variables are unknown, but as shown in Figure 2, we assumed that confounding factors are those occurring before ACE and outcome, and further that mediating factors are those occurring before outcome.

### Covariates

The covariates were sex (male or female), age group (20-39 or 40-64 years), and educational attainment (junior high school, high school, college, university or graduate school, or others). These data were collected using a questionnaire.

### Statistical Analysis

Data were analyzed from July 2023 to April 2024. We used causal mediation analysis using the paramed command in Stata to investigate the association between ACE, each mediator, and elder abuse (Figure 2).^36^ We calculated the direct and indirect effect estimates using logistic regression analyses and estimated the ORs and their 95% CIs. Natural direct effect estimate (NDE) represents the effect of the exposure on the outcome that is not mediated by the mediator. Natural indirect effect estimate (NIE) represents the effect of the exposure on the outcome that is mediated by the mediator.^37^ Furthermore, the total effect estimate, which is the sum of the indirect and direct effects, is represented. The total effect estimate of the exposure on the outcome can be decomposed into the sum of the NDE and NIE. Moreover, we estimated the proportion mediated (PM), which is the proportion of the mediating effect estimate of the total effect estimate divided by the indirect and total effect estimates. We used following formula to calculate PM: [OR_NDE_ (OR_NIE_ − 1)] / [OR_NDE_ × OR_NIE_ − 1].^38^

In the mediation analysis, exposure X leading to outcome Y as NDE and mediator M leading to outcome Y were analyzed using logistic regression, and exposure X leading to mediator M was analyzed using linear regression. A logistic regression analysis with all variables entered was also performed, and the results are presented in eTable 1 in Supplement 1. The results of the mediation analysis using the crude model are presented in eTable 2 in Supplement 1. In addition, the analysis results without excluding participants aged 65 years and older are in eTable 3 in Supplement 1, and the analysis stratifying outcomes into physical and psychological abuse are in eTable 4 in Supplement 1. eTable 5 in Supplement 1 shows the association between ACEs of continuous values and elder abuse with confounding factors. These data did not contain missing data because the online survey needed to complete questions to progress answering questionnaire. A 2-sided *P* < .05 was considered statistically significant. All statistical analyses were performed using Stata statistical software version 17/IC (StataCorp).

## Results

### Participants’ Characteristics

Table 1 shows the characteristics of the 13 318 study participants. The mean (SD) age of the participants was 41.1 (12.1) years, and 6634 (49.8%) were female. Among the participants, 1133 (8.5%) had perpetrated abuse against older adults. The incidences of physical and psychological abuse were 5.99% and 7.97%, respectively. In total, 6556 participants (49.2%) had 0 ACEs, 4875 (36.6%) had 1 ACE, and 1887 (14.2%) had 2 or more ACEs.

**Table 1.** Descriptive Characteristics of Study Participants by Number of ACEs

Characteristic	Participants, No. (%)			
	0 ACEs (n = 6556)	1 ACE (n = 4875)	≥2 ACEs (n = 1887)	Total (N = 13 318)
Abuse of older people				
No	6334 (96.6)	4360 (89.4)	1491 (79.0)	12 185 (91.5)
Yes	222 (3.4)	515 (10.6)	396 (21.0)	1133 (8.5)
Sex				
Male	3150 (48.0)	2637 (54.1)	897 (47.5)	6684 (50.2)
Female	3406 (52.0)	2238 (45.9)	990 (52.5)	6634 (49.8)
Age group, y				
20-39	3204 (48.9)	2585 (53.0)	949 (50.3)	6738 (50.6)
40-64	3352 (51.1)	2290 (47.0)	938 (49.7)	6580 (49.4)
Educational attainment				
Junior high school	45 (0.7)	70 (1.4)	57 (3.0)	172 (1.3)
High school	1139 (17.4)	1006 (20.6)	578 (30.6)	2723 (20.4)
College	1456 (22.2)	1100 (22.6)	413 (21.9)	2969 (22.3)
University or graduate school	3898 (59.5)	2681 (55)	821 (43.5)	7400 (55.6)
Other	18 (0.3)	18 (0.4)	18 (1.0)	54 (0.4)
Living arrangement				
With someone	5397 (82.3)	3875 (79.5)	1443 (76.5)	10715 (80.5)
Alone	1159 (17.7)	1000 (20.5)	444 (23.5)	2603 (19.5)
Employment status				
Working	5479 (83.6)	4198 (86.1)	1591 (84.3)	11 268 (84.6)
Unemployed	1077 (16.4)	677 (13.9)	296 (15.7)	2050 (15.4)
Marital status				
Not married	2176 (33.2)	1902 (39.0)	784 (41.5)	4862 (36.5)
Married	4380 (66.8)	2973 (61.0)	1103 (58.5)	8456 (63.5)
Equivalent income				
Middle or high income	4542 (69.3)	3191 (65.5)	1144 (60.6)	8877 (66.7)
Low income	2014 (30.7)	1684 (34.5)	743 (39.4)	4441 (33.3)
Self-rated health				
Not good	2363 (36.0)	2074 (42.5)	973 (51.6)	5410 (40.6)
Good	4193 (64.0)	2801 (57.5)	914 (48.4)	7908 (59.4)
Depression				
Never	5869 (89.5)	4139 (84.9)	1333 (70.6)	11 341 (85.2)
Having in the past or present	687 (10.5)	736 (15.1)	554 (29.4)	1977 (14.8)
Psychological disorder except depression				
Never	6044 (92.2)	4309 (88.4)	1435 (76.0)	11 788 (88.5)
Having in the past or present	512 (7.8)	566 (11.6)	452 (24.0)	1530 (11.5)
Alcohol consumption				
Currently not drinking	2316 (35.3)	1867 (38.3)	639 (33.9)	4822 (36.2)
Currently drinking	4240 (64.7)	3008 (61.7)	1248 (66.1)	8496 (63.8)
Going out at least once a month				
No	39 (0.6)	71 (1.5)	28 (1.5)	138 (1.0)
Yes	6517 (99.4)	4804 (98.5)	1859 (98.5)	13 180 (99.0)

Abbreviation: ACE, adverse child experience.

### Logistic Regression Analysis

Table 2 shows the ORs for the association between elder abuse and ACEs, with covariates. Compared with participants with 0 ACEs, the ORs for abuse were 3.22 (95% CI, 2.74-3.79) for those with 1 ACE and 7.65 (95% CI, 6.41-9.13) for those with 2 or more ACEs.

**Table 2.** Results of Logistic Regression Analysis With All Covariates

Variable	Participants, No. (N = 13 318)	Crude analysis		Controlled covariates	
		OR (95% CI)	*P* value	OR (95% CI)	*P* value
No. of adverse childhood experiences					
0	6556	1 [Reference]	NA	1 [Reference]	NA
1	4875	3.37 (2.87-3.96)	<.001	3.22 (2.74-3.79)	<.001
≥2	1887	7.58 (6.37-9.02)	<.001	7.65 (6.41-9.13)	<.001
Sex					
Male	6684	NA	NA	1 [Reference]	<.001
Female	6634	NA	NA	0.51 (0.45-0.59)	
Age group, y					
20-39	6738	NA	NA	1 [Reference]	<.001
40-64	6580	NA	NA	0.78 (0.69-0.89)	
Educational attainment					
Junior high school	172	NA	NA	1 [Reference]	NA
High school	2723	NA	NA	0.86 (0.54-1.38)	.54
College	2969	NA	NA	0.84 (0.52-1.35)	.48
University or graduate school	7400	NA	NA	0.84 (0.53-1.33)	.45
Other	54	NA	NA	1.06 (0.43-2.61)	.91

Abbreviations: NA, not applicable; OR, odds ratio.

### Causal Mediation Analysis

Table 3 shows the ORs and proportion of mediating effects of each mediator between ACEs and elder abuse for the NDE, NIE, total effect estimate, and PM. Most associations were explained by psychological factors, such as depression (OR, 1.13; 95% CI, 1.11-1.14; PM, 18.6%), psychological disorders other than depression (OR, 1.12; 95% CI, 1.10-1.14; PM, 17.3%), and self-rated health (OR, 1.04; 95% CI, 1.03-1.05; PM, 6.0%). In contrast, a relatively small proportion of the association was mediated by living arrangement, marital status, equivalent income, and going out at least once a month. The mediators with less than 1% PM were employment status and alcohol consumption.

**Table 3.** Decomposition of the Total Effect Estimate of the Association Between Adverse Childhood Experiences and Elder Abuse Into Direct and Indirect Effect Estimates Using Causal Mediation Analysis (N = 13 318)

Mediators	Natural direct effect estimate		Natural indirect effect estimate		Total effect estimate		PM, %
	OR (95% CI)	*P* value	OR (95% CI)	*P* value	OR (95% CI)	*P* value	
Living arrangement	2.72 (2.50-2.96)	.04	1.01 (1.001-1.011)	.002	2.74 (2.51-2.98)	.04	1.6
Employment status	2.69 (2.39-3.03)	<.001	1.00 (0.997-1.001)	.28	2.69 (2.39-3.02)	<.001	0.0
Marital status	2.70 (2.18-3.34)	<.001	1.02 (1.01-1.03)	<.001	2.75 (2.22-3.41)	<.001	3.1
Equivalent income	2.70 (2.24-3.25)	<.001	1.01 (1.007-1.020)	<.001	2.73 (2.27-3.30)	<.001	1.6
Self-rated health	2.65 (2.34-3.02)	<.001	1.04 (1.03-1.05)	<.001	2.77 (2.44-3.15)	<.001	6.0
Depression	2.32 (1.89-2.86)	<.001	1.13 (1.11-1.14)	<.001	2.62 (2.13-3.21)	<.001	18.6
Psychological disorder except depression	2.35 (1.93-2.86)	<.001	1.12 (1.10-1.14)	<.001	2.63 (2.16-3.21)	<.001	17.3
Alcohol consumption	2.73 (2.50-2.98)	<.001	1.00 (0.999-1.001)	.94	2.73 (2.50-2.98)	<.001	0.0
Going out at least once a month	2.73 (2.45-3.05)	<.001	1.01 (1.00-1.011	<.001	2.75 (2.46-3.07)	<.001	1.6

Abbreviations: OR, odds ratio; PM, proportion mediated.

## Discussion

In this cross-sectional study, higher numbers of ACEs were clearly associated with perpetration of abuse against older adults. This finding suggests that the intergenerational chain of abuse is associated not only with child abuse but also with the perpetration of elder abuse. Depression, mental illnesses other than depression, and self-rated health had large NIEs as mediators. To the best of our knowledge, this is the first study to elucidate the association between ACEs and elder abuse and its mediators.

One reason for this result may be continued family discord. Several studies^1,39,40,41^ have shown that physical abuse and neglect of one’s children is more likely among individuals with higher cumulative ACEs. Furthermore, corporal punishment is more likely to be a social and cultural factor than a personality trait.^15^ Unless individuals who experience corporal punishment as children question such an environment or have the experience to escape it, they may end up becoming abusers, such as when their parents get older and need care. Also, being in such an environment may cause parents to view corporal punishment, or violence, as legitimate.^42^ According to Japanese government reports,^43,44^ most elder abuse occurs between the older adults being cared for and those who care for them.

Furthermore, we found that psychological factors, such as depression, mental illness other than depression, and self-rated health, were large indirect mediators between ACEs and elder abuse perpetrators. In this study, how long these psychological factors existed with the perpetrators was unclear. For example, it is unknown whether depression is caused by ACEs or by other factors in later life. Studies have shown that ACEs can affect depression and well-being in old age.^20^ Moreover, a 2022 systematic review by Misiak et al^45^ reported that psychosocial stress in childhood may have a lasting impact on biological dysregulation captured by allostatic load, which is one of the concepts of adopting an environment. Those exposed to ACEs had a higher allostatic load index. Therefore, when faced with a difficult situation, recipients of abuse may not be able to adapt well and may become stressed, eventually leading to them committing violence. In addition, it is has been reported that harsh punishment, including corporal punishment, may lead to mental health problems or depression in adolescence, and even lower the self-control of those who experience it.^46,47,48^ Also, those negative outcomes on mental health may continue to manifest throughout adolescence and adulthood.^11^

There is research of elder abuse using large epidemiological data sources in Japan. According to those studies,^27,29^ the rates of occurrence of physical abuse were 0.9% to 1.26% and those of psychological abuse were 10.1% to 11.1%. In the present study, the incidences of physical and psychological abuse were 5.99% and 7.97%, respectively. Our study differs from previous ones in 3 ways: first, previous studies used data from self-administered mail surveys; second, they inquired about the circumstances of experiencing abuse among older individuals; and third, they targeted older adults, whereas this study focuses on adults in general. Although direct comparison is challenging because of various biases and factors, considering these differences is beneficial.

### Strengths and Limitations

This study’s strength is its focus on elder abuse perpetration, which is often overlooked in research about ACEs. Gathering data on both recipients and perpetrators of abuse is challenging. However, we succeeded in collecting and analyzing such data, enabling us to design a study that explores the background and individual factors of perpetrators, advancing research in this field.

This study also has several limitations. First, using a retrospective method to assess ACEs may induce recall biases, because retrospective self-reports often differ from contemporaneous ones and reflect current psychological states.^49,50^ The questionnaire survey might underestimate actual cases owing to reporting bias, although online surveys may capture more-accurate data by reducing social desirability bias.^51^ Second, our assessment of elder abuse perpetration lacks validation, because there is no standard measure for such incidents. We included specific actions to evaluate incidents from multiple angles, but future research should address the relationships among the individuals involved. Third, we did not collect data on financial abuse, sexual abuse, or neglect, thus limiting our analysis to physical and psychological abuse. Future studies should cover all 5 types of elder abuse.^52,53^ In addition, excluding participants aged 65 years and older omits data on violence from spouses and partners, and we also lack racial data for participants. Fourth, we did not consider social or community factors, which can influence the cycle of violence. Future analyses should include these factors. Fifth, mediators were analyzed individually, not considering intermediator effects. Future studies should use mediator-wide analyses.^54^ Sixth, because this is a cross-sectional study, causal relationships cannot be established. In addition, the time frames for exposure variables, intermediate factors, and outcomes are ambiguous. Thus, a longitudinal study using panel data, which considers the time axis, is necessary. Addressing these limitations is essential for generating evidence that can inform policies to prevent violent acts by perpetrators.

## Conclusions

In this cross-sectional study, adults with a higher number of ACEs had an increased risk of committing elder abuse, thus perpetuating the intergenerational chain of abuse. These findings suggest that the cycle of violence may extend to any vulnerable group, not only to children but also older adults. Despite the limitations, this study showed results indicating an even longer negative effect of ACEs than previously understood. Further research is warranted to prevent ACEs and disrupt the intergenerational cycle of violence and abuse.

## Notes

Supplement 1. **eTable 1.** Results of the logistic regression analysis with all variables **eTable 2.** Causal mediation analysis of the effect of each mediator on the elder abuse with crude results **eTable 3.** Results from logistic regression analysis with all variables including 65 and older people **eTable 4.** Results of logistic regression analysis for each of the physical and psychological abuse outcomes **eTable 5.** Results from logistic regression analysis with all covariates with ACE as continuous values

Supplement 2.
Data Sharing Statement
