# Supplementary material for: Intergenerational Chain of Violence, Adverse Childhood Experiences, and Elder Abuse Perpetration
Source: JAMA Netw Open. 2024 Sep 27;7(9):e2436150. doi: 10.1001/jamanetworkopen.2024.36150 (PMC11437385; doi:10.1001/jamanetworkopen.2024.36150)
Supplement: Supplement 1. — eTable 1. Results of the logistic regression analysis with all variables eTable 2. Causal mediation analysis of the effect of each mediator on the elder abuse with crude results eTable 3. Results from logistic regression analysis with all variables including 65 and older people eTable 4. Results of logistic regression analysis for each of the physical and psychological abuse outcomes eTable 5. Results from logistic regression analysis with all covariates with ACE as continuous values [file jamanetwopen-e2436150-s001.pdf]

Supplemental Online Content

Koga C, Tsuji T, Hanazato M, Tabuchi T. Intergenerational chain of violence, adverse childhood experiences, and elder abuse perpetration. *JAMA Netw Open*. 2024;7(9):e2436150. doi:10.1001/jamanetworkopen.2024.36150

eTable 1. Results of the logistic regression analysis with all variables

eTable 2. Causal mediation analysis of the effect of each mediator on the elder abuse with crude results

eTable 3. Results from logistic regression analysis with all variables including 65 and older people

eTable 4. Results of logistic regression analysis for each of the physical and psychological abuse outcomes

eTable 5. Results from logistic regression analysis with all covariates with ACE as continuous values

This supplemental material has been provided by the authors to give readers additional information about their work.

© 2024 Koga C et al. *JAMA Network Open.*

eTable 1. Results of the logistic regression analysis with all variables.

	n	OR (95%CI)	p
**Number of ACE**			
None	6,556	1.00	
One	4,875	2.86 (2.420–3.386)	<0.001
More than two	1,887	5.05 (4.185–6.088)	<0.001
**Sex**			
Male	6,684	1.00	
Female	6,634	0.52 (0.451–0.603)	<0.001
**Age**			
20-39	6,738	1.00	
40-64	6,580	0.92 (0.803–1.058)	0.25
**Educational attainment**			
Junior High School	172	1.00	
High School	2,723	1.46 (0.877–2.417)	0.15
College	2,969	1.41 (0.844–2.342)	0.19
University/Graduate school	7,400	1.57(0.951–2.585)	0.08
Others	54	1.37 (0.497–3.791)	0.54
**Living arrangement**			
Alone with someone	10,715	1.00	
Living alone	2,603	0.86 (0.715–1.034)	0.11
**Employment status**			
Working	11,268	1.00	

© 2024 Koga C et al. *JAMA Network Open.*

Unemployed	2,050	0.95 (0.776–1.174)	0.66
**Marital status**			
Married	4,862	1.00	
Not married	8,456	0.66 (0.563–0.776)	<0.001
**Equivalent income**			
Middle or high income	8,877	1.00	
Low income	4,441	1.33 (1.152–1.529)	<0.001
**Self-rated health**			
Not good	5,410	1.00	
Good	7,908	0.80 (0.699–0.921)	0.002
**Depression**			
Never	11,341	1.00	
Having in the past or present	1,977	2.33 (1.975–2.752)	<0.001
**Psychological disorder except depression**			
Never	11,788	1.00	
Having in the past or present	1,530	3.29 (2.776–3.908)	<0.001
**Alcohol consumption**			
Currently not drinking	4,822	1.00	
Currently Drinking	8,496	0.99 (0.863–1.143)	0.92
**Going out at least once a month**			
No	138	1.00	
Yes	13,180	0.28 (0.185–0.426)	<0.001

Abbreviations: CI, confidence interval; OR, odds ratio; ACEs, adverse childhood experiences

© 2024 Koga C et al. *JAMA Network Open.*

eTable 2. Causal mediation analysis of the effect of each mediator on the elder abuse with crude results

	Natural direct effect				Natural indirect effect				Total effect			
	Crude		Controlled ovariates		Crude		Controlled ovariates		Crude		Controlled ovariates	
Mediators	OR (95%CI)	p	OR (95%CI)	p	OR (95%CI)	p	OR (95%CI)	p	OR (95%CI)	p	OR (95%CI)	p
Living arrangement												
	2.69 (2.470–2.922)	<0.001	2.72 (2.495–2.964)	0.04	1.01 (1.003–1.013)	<0.001	1.01 (1.001–1.011)	0.002	2.71 (2.490–2.946)	<0.001	2.74 (2.510–2.982)	0.04
Employment status												
	2.70 (2.482–2.934)	<0.001	2.69 (2.393–3.027)	<0.001	1.00 (0.999–1.004)	0.16	1.00 (0.997–1.001)	0.28	2.70 (2.486–2.939)	<0.001	2.69 (2.390–3.024)	<0.001
Marital status												
	2.65 (2.115–3.317)	<0.001	2.70 (2.175–3.342)	<0.001	1.03 (1.017–1.035)	<0.001	1.02 (1.013–1.029)	<0.001	2.72 (2.169–3.403)	<0.001	2.75 (2.220–3.411)	<0.001
Equivalent income												
	2.67 (2.246–3.171)	<0.001	2.70 (2.239–3.254)	<0.001	1.02 (1.010–1.024)	<0.001	1.01 (1.007–1.020)	<0.001	2.71 (2.284–3.225)	<0.001	2.73 (2.268–3.296)	<0.001
Self-rated health												
	2.62 (2.311–2.976)	<0.001	2.65 (2.336–3.018)	<0.001	1.04 (1.032–1.056)	<0.001	1.04 (1.031–1.054)	<0.001	2.74 (2.412–3.106)	<0.001	2.77 (2.435–3.146)	<0.001
Depression												
	2.30 (1.849–2.868)	<0.001	2.32 (1.892–2.855)	<0.001	1.13 (1.109–1.147)	<0.001	1.13 (1.107–1.144)	<0.001	2.60 (2.086–3.235)	<0.001	2.62 (2.129–3.213)	<0.001
Psychological disorder except depression												
	2.32 (1.873–2.864)	<0.001	2.35 (1.925–2.864)	<0.001	1.13 (1.107–1.144)	<0.001	1.12 (1.103–1.139)	<0.001	2.61 (2.107–3.224)	<0.001	2.63 (2.157–3.211)	<0.001
Alcohol consumption												

© 2024 Koga C et al. *JAMA Network Open.*

	2.70 (2.487–2.939)	<0.001	2.73 (2.504–2.978)	<0.001	1.00 (1.000–1.000)	0.89	1.00 (0.999–1.001)	0.94	2.70 (2.487–2.939)	<0.001	2.73 (2.504–2.978)	<0.001
Going out at least once a month												
	2.70 (2.405–3.023)	<0.001	2.73 (2.447–3.049)	<0.001	1.01 (1.004–1.013)	<0.001	1.01 (1.003–1.011)	<0.001	2.72 (2.425–3.049)	<0.001	2.75 (2.463–3.070)	<0.001

Abbreviations: CI, confidence interval; OR, odds ratio; PM, proportion mediated; ACEs, adverse childhood experiences

eTable 3. Results from logistic regression analysis with all variables including 65 and older people

	n	OR (95%CI)	p
**Number of ACE**			
None	9305	1.00	
One	6,413	2.45 (2.118–2.844)	<0.001
More than two	2,320	4.32 (3.656–5.110)	<0.001
**Sex**			
Male	16,721	1.00	
Female	1,317	0.57 (0.496–0.646)	<0.001
**Age**			
20-39	6,738	1.00	
40-64	6,580	0.91 (0.793–1.040)	0.25
Over 65	4,720	0.65 (0.524–0.798)	<0.001
**Educational attainment**			
Junior High School	269	1.00	
High School	4,435	1.53 (0.956–2.440)	0.08
College	3,841	1.50 (0.934–2.407)	0.09
University/Graduate school	9,433	1.70(1.068–2.700)	0.03
Others	60	1.76 (0.682–4.558)	0.24
**Living arrangement**			
Alone with someone	14,548	1.00	
Living alone	3,490	0.81 (0.678–0.959)	0.02
**Employment status**			

© 2024 Koga C et al. *JAMA Network Open.*

Working	12,559	1.00	
Unemployed	5,479	0.95 (0.798–1.132)	0.57
**Marital status**			
Married	5,973	1.00	
Not married	12,065	0.68 (0.587–0.797)	<0.001
**Equivalent income**			
Middle or high income	11,541	1.00	
Low income	6,497	1.34 (1.174–1.530)	<0.001
**Self-rated health**			
Not good	7,177	1.00	
Good	10,861	0.77 (0.683–0.877)	<0.001
**Depression**			
Never	15,704	1.00	
Having in the past or present	2,334	2.26 (1.936–2.632)	<0.001
**Psychological disorder except depression**			
Never	16,354	1.00	
Having in the past or present	1,684	3.26 (2.772–3.823)	<0.001
**Alcohol consumption**			
Currently not drinking	6,558	1.00	
Currently Drinking	11,480	1.01 (0.886–1.146)	0.90
**Going out at least once a month**			
No	168	1.00	
Yes	17,870	0.32 (0.215–0.474)	<0.001

© 2024 Koga C et al. *JAMA Network Open.*

Abbreviations: CI, confidence interval; OR, odds ratio; ACEs, adverse childhood experiences

© 2024 Koga C et al. *JAMA Network Open.*

eTable 4. Results of logistic regression analysis for each of the physical and psychological abuse outcomes.

		Physical abuse		Psychological abuse	
	n	OR (95%CI)	p	OR (95%CI)	p
**Number of ACE**					
None	6,556	1.00		1.00	
One	4,875	3.74 (3.008–4.656)	<0.001	2.90 (2.438–3.452)	<0.001
More than two	1,887	6.89 (5.442–8.736)	<0.001	5.16 (4.258–6.260)	<0.001
**Sex**					
Male	6,684	1.00		1.00	
Female	6,634	0.37 (0.312–0.446)	<0.001	0.51 (0.439–0.591)	<0.001
**Age**					
20-39	6,738	1.00		1.00	
40-64	6,580	0.58 (0.488–0.683)	<0.001	0.93 (0.808–1.072)	0.32
**Educational attainment**					
Junior High School	172	1.00		1.00	
High School	2,723	1.43 (0.787–2.590)	0.24	1.38 (0.830–2.288)	0.22
College	2,969	1.27 (0.695–2.314)	0.44	1.36 (0.813–2.258)	0.24
University/Graduate school	7,400	1.59(0.883–2.848)	0.12	1.40(0.851–2.316)	0.18
Others	54	1.50 (0.482–4.674)	0.48	1.11 (0.388–3.195)	0.84
**Living arrangement**					
Alone with someone	10,715	1.00		1.00	
Living alone	2,603	1.02 (0.818–1.270)	0.86	0.86 (0.713–1.040)	0.12
**Employment status**					

© 2024 Koga C et al. *JAMA Network Open.*

Working	11,268	1.00		1.00	
Unemployed	2,050	0.80 (0.616–1.050)	0.11	0.96 (0.774–1.183)	0.69
**Marital status**					
Married	4,862	1.00		1.00	
Not married	8,456	0.91 (0.748–1.103)	0.33	0.67 (0.569–0.791)	<0.001
**Equivalent income**					
Middle or high income	8,877	1.00		1.00	
Low income	4,441	1.30 (1.097–1.539)	0.002	1.31 (1.129–1.510)	<0.001
**Self-rated health**					
Not good	5,410	1.00		1.00	
Good	7,908	0.94 (0.800–1.113)	0.49	0.80 (0.693–0.920)	0.002
**Depression**					
Never	11,341	1.00		1.00	
Having in the past or present	1,977	2.84 (2.337–3.454)	<0.001	2.31 (1.950–2.738)	<0.001
**Psychological disorder except depression**					
Never	11,788	1.00		1.00	
Having in the past or present	1,530	3.71 (3.039–4.526)	<0.001	3.32 (2.789–3.955)	<0.001
**Alcohol consumption**					
Currently not drinking	4,822	1.00		1.00	
Currently Drinking	8,496	0.92 (0.775–1.081)	0.30	0.98 (0.852–1.136)	0.82
**Going out at least once a month**					
No	138	1.00		1.00	
Yes	13,180	0.22 (0.143–0.352)	<0.001	0.32 (0.205–0.484)	<0.001

© 2024 Koga C et al. *JAMA Network Open.*

Abbreviations: CI, confidence interval; OR, odds ratio; ACEs, adverse childhood experiences

© 2024 Koga C et al. *JAMA Network Open.*

eTable 5. Results from logistic regression analysis with all covariates with ACE as continuous values

		Crude		Controlled covariates	
	n	OR (95%CI)	p	OR (95%CI)	p
Number of ACEs					
(Continuous value)	13,318	2.71 (2.493–2.944)	<0.001	2.74 (2.512–2.981)	<0.001
Sex					
Man	6,684			1.00	
Women	6,634			0.51 (0.443–0.578)	<0.001
Age					
20-39	6,738			1.00	
40-64	6,580			0.78 (0.683–0.883)	<0.001
Educational attainment					
Junior High School	172			1.00	
High School	2,723			0.86 (0.539–1.387)	0.55
College	2,969			0.85 (0.529–1.370)	0.51
University/Graduate school	7,400			0.84 (0.531–1.343)	0.48
Others	54			1.04 (0.421–2.588)	0.93

Abbreviations: CI, confidence interval; OR, odds ratio; ACEs, adverse childhood experiences

© 2024 Koga C et al. *JAMA Network Open.*
