# Supplementary material for: Intergenerational Chain of Violence, Adverse Childhood Experiences, and Elder Abuse Perpetration
Source: JAMA Netw Open. 2024 Sep 27;7(9):e2436150. doi: 10.1001/jamanetworkopen.2024.36150 (PMC11437385; doi:10.1001/jamanetworkopen.2024.36150)
Supplement: Supplement 2. — Data Sharing Statement [file jamanetwopen-e2436150-s002.pdf]

Data Sharing Statement

Koga. Intergenerational Chain of Violence, Adverse Childhood Experiences, and Elder Abuse Perpetration. *JAMA Netw Open*. Published September 27, 2024.
doi:10.1001/jamanetworkopen.2024.36150

Data
Data available: No

Additional Information
Explanation for why data not available: If it is for academic use, the dataset is available by applying for a research proposal.
